# Hold that Thought: Organochlorines May Alter Infant Attention Skills

**Published:** 2008-05

**Authors:** Tanya Tillett

From the mid-1940s on, a group of synthetic chemicals known as organochlorines (OCs) were used in industry and as pesticides. Because of evidence of human and environmental risks from exposure to some of these chemicals, the Environmental Protection Agency in the 1970s banned the use of two—the pesticide DDT and a class of industrial chemicals known as polychlorinated biphenyls (PCBs). Despite the ban of more than 30 years, residues of these persistent, bioaccumulative chemicals are still found in human tissue. Now researchers have found evidence of an association between poor attention skills in early infancy and low-level prenatal exposure to PCBs and *p,p*′-DDE, the chief metabolite of DDT **[*EHP* 116:666–673; Sagiv et al.]**.

Previous studies have shown associations between PCB exposure and attention deficits in adults and school-age children. In the current study, the authors sought to investigate whether these associations could be detected in early infancy using the Neonatal Behavioral Assessment Scale (NBAS) to assess infants’ visual and auditory stimuli responses, motor tone and motor activity levels, and ability to regulate crying, alert, and sleep states. These behavioral items identify the infant’s capacity for attention as well as abilities potentially associated with attention, such as state regulation and motor maturity.

The present study included infants born between 1993 and 1998 to mothers who lived near a PCB-contaminated harbor in New Bedford, Massachusetts. The NBAS was administered to infants twice: between 1 and 3 days after birth and between 5 and 22 days after birth. The researchers also administered questionnaires to mothers of the infants to collect demographic data on household income; medical and reproductive histories; drug, alcohol, and tobacco use; race and ethnicity; and occupational and exposure histories pertinent to outcomes of interest.

To determine OC levels in the infants, the researchers analyzed infant cord serum samples, taken at birth, for 51 PCB congeners and *p,p*′-DDE. Of the total serum samples, 96% had detectable DDE levels; depending on the PCB congener, anywhere from fewer than 1% to 91% of the infants had detectable serum levels. For the 542 infants that completed both exams, statistical analysis revealed a decline in scores of attention with increased serum levels of DDE and of PCBs. The exposure-associated declines were less pronounced for abilities associated with attention, including state- and motor-associated outcomes.

The authors acknowledge the uncertainty of the NBAS in accurately predicting attention behaviors that may occur later in childhood. Future studies should examine whether the attention deficits observed in the current study in association with organochlorine exposure persist into childhood.

## Figures and Tables

**Figure:**
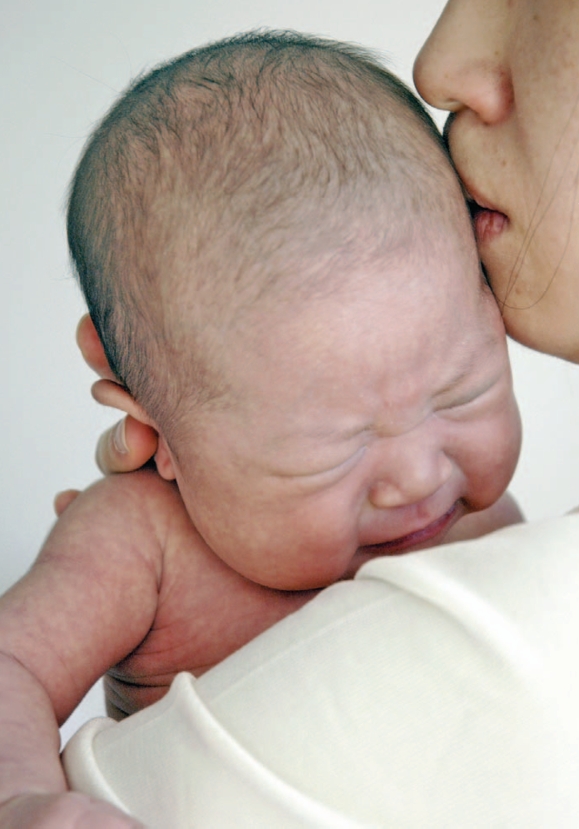
Soothability can predict later attention skills

